# Effects of Zinc on Metallicolous and Non-Metallicolous Populations of *Noccaea caerulescens*

**DOI:** 10.3390/plants14131975

**Published:** 2025-06-27

**Authors:** Anna D. Kozhevnikova, Alexander V. Kartashov, Ilya V. Seregin

**Affiliations:** K.A. Timiryazev Institute of Plant Physiology, Russian Academy of Sciences, Botanicheskaya st., 35, Moscow 127276, Russia; botanius@yandex.ru (A.V.K.); ilya_seregin@mail.ru (I.V.S.)

**Keywords:** *Noccaea caerulescens*, Wilwerwiltz population, Prayon population, metal accumulation, mineral nutrition, metal translocation, metal uptake, zinc

## Abstract

The aim of this study was to evaluate whether intraspecific differences in zinc (Zn) tolerance and accumulation in the hyperaccumulator *Noccaea caerulescens* are linked to Zn-induced changes in transpiration and mineral composition. At 500 µM Zn in the nutrient solution, a decrease in the root and shoot biomass, the water content in roots, and the contents of photosynthetic pigments in shoots was observed only in the non-metallicolous population Wilwerwiltz, whereas in the calamine population Prayon, root growth was stimulated. Zinc-induced impairment of mineral nutrition was greater in Wilwerwiltz than in Prayon, which determined the manifestation of Zn toxicity in Wilwerwiltz. The absence of signs of Zn toxicity and the stimulation of root growth in Prayon may be due to lower Zn accumulation in Prayon than in Wilwerwiltz, as well as more effective mechanisms of Zn detoxification. The higher Zn content in the shoots and, in particular, in the water-storage cells of the leaf epidermis in Wilwerwiltz compared to Prayon may be partly due to the higher transpiration rate in Wilwerwiltz, at least at 500 µM Zn. These findings suggest that the metallicolous population maintains better control over Zn accumulation, which may be a part of the adaptive response to Zn-enriched media.

## 1. Introduction

Mineral elements play important roles in the metabolism of plants, which serve as the sources of their entry into the food chain. Zinc (Zn) is a microelement whose optimal level for most plants ranges from 30 to 200 μg Zn per gram of dry weight [1,2,3]. This element forms complexes with DNA and RNA, altering their stability, and is involved in the metabolism of proteins, lipids, nucleic acids, and carbohydrates [1,4,5,6,7]. Zinc plays an important role in maintaining membrane stability [5,8], is necessary for hormone regulation [5,6], and is also the only element found in all six major enzyme classes (oxidoreductases, transferases, hydrolases, lyases, isomerases, and ligases) [3,6,9]. It has been estimated that Zn is used as a ligand by more than 10% of proteins, which comprise the so-called zinc proteome [3]. Zinc-finger-domain-containing proteins are involved in the binding of DNA to transcription factors and protein-protein interactions [10], thus functioning as key transcriptional regulators in plant responses to abiotic [11] and biotic stresses [12].

The toxic effects of Zn on a wide variety of physiological processes are often observed at supra-optimal Zn concentrations in plants [2]. Under excess Zn, the manifestations of its toxic effects include changes in the contents of macro- and microelements [2,13,14,15,16,17], a decrease in the water content and transpiration rate [2,15], the inactivation of multiple enzymes [2], DNA damage [18], changes in the fatty acid composition of memsbrane lipids [19], disruption of membrane integrity and permeability [20], inhibition of cell division and elongation [14,15,18,21], changes in the architecture of the root system [17], and decreases in the contents of photosynthetic pigments and the activity of photosystem II [2,5,22,23]. This results in leaf chlorosis and a decrease in the rate of photosynthesis, which, in turn, leads to growth inhibition, disruption of morphogenesis, and a decrease in plant productivity [2,21,22,23,24].

Symptoms of toxicity start to appear at different metal contents in various plant species, which reflects their dissimilar metal tolerances and accumulation capacities [25]. In nature, there are two contrasting groups of plants: excluders, in which metals accumulate mainly in the root system, and hyperaccumulators, in which they accumulate mainly in the aboveground organs [26]. Currently, more than 800 species of metal hyperaccumulator plants capable of accumulating large amounts of metals in shoots without visible disruption of metabolism and growth processes are known [25,27,28]. Some of them are considered promising species for phytoremediation [29], phytomining [30], and co-cultivation with agricultural species [31]. The phenomenon of Zn hyperaccumulation was first described in 1865 for *Noccaea caerulescens* by J. Sachs [32]. At present, more than 20 plant species from nine families, mainly from Brassicaceae, are classified as Zn hyperaccumulators capable of accumulating more than 3000 μg Zn per gram shoot dry weight under natural growth conditions [8,27,28,33,34].

*Noccaea caerulescens*, a pseudometallophyte and a hyperaccumulator of Zn, nickel (Ni), and cadmium (Cd), is a model species for studying various aspects of hyperaccumulation and the associated metal hypertolerance [35,36]. Zn hyperaccumulation has been confirmed as a constitutive trait in *N. caerulescens*, since it was shown for the plants from metalliferous (metal-enriched) and non-metalliferous soils [37]. The capacity for metal hyperaccumulation could have evolved in plants growing on metalliferous soils as well as in plants growing on non-metalliferous soils, with subsequent colonization of metalliferous soils and an increase in plant metal tolerance [25,38,39]. Among metalliferous soils, serpentine soils, derived from ultramafic rocks enriched in Ni, cobalt (Co), magnesium (Mg), manganese (Mn), iron (Fe), and chromium (Cr), as well as calamine soils, enriched in Zn, Cd, and lead (Pb), are of greatest interest [25,39,40,41,42,43]. A comprehensive study of the demographic history of *N. caerulescens* in Western Europe suggested that the large-scale genetic structure of its populations preceded the local adaptation to metalliferous sites, and multiple independent events involving the colonization of metalliferous soils after the isolation of genetic subunits were followed by local adaptation from standing variation and phenotypic convergence in similar environments [44].

Although Zn hyperaccumulation and hypertolerance are constitutive in *N. caerulescens*, significant differences in Zn tolerance and Zn accumulation capacities have been found between plants from different populations, which is associated with different edaphic origins and/or phylogeographic patterns [25,40,41,42,45,46]. Representatives of several populations of *N. caerulescens* are capable of accumulating up to 30,000–50,000 μg Zn g^−1^ shoot dry weight without showing symptoms of toxicity [47,48]. The analysis of 28 hydroponically grown *N. caerulescens* populations revealed that the variation in the capacity to accumulate Zn in shoots between the populations from calamine soils was significantly greater than that between the populations from ultramafic soils or between the populations from non-metalliferous soils [25]. When grown in soil (at 2000 μg g^−1^ Zn), the plants of the Prayon population, which naturally grow on calamine soils in Belgium, accumulated less Zn in their leaves than did the plants of the Wilwerwiltz population, which naturally grow on non-metalliferous soils in Luxembourg [49]. The average value of the translocation factor, i.e., the ratio of the Zn content in shoots to its content in roots, of the 19 populations studied was the highest in plants of the calamine populations Prayon and Plombières and non-metallicolous populations Wilwerwiltz and Jean Arsac [42]. Plant Zn tolerance, as assessed by the Zn concentration in the nutrient solution at which a complete cessation of root growth was observed, was greater for the Prayon population (5000 μM Zn) compared to the Wilwerwiltz population (4250 μM Zn) [25]. These contrasting differences revealed between the plants from these two populations determined the relevance of their further examination within the framework of the current study.

The reasons for intraspecific differences in metal tolerance and accumulation capacity, even in model plant species, are not fully understood. In general, the mechanisms of metal hyperaccumulation can potentially be controlled at the levels of metal absorption from the soil, radial and long-distance transport, and accumulation in leaves [36,50,51,52,53]. In different populations of *N. caerulescens*, the mechanisms of metal uptake [54], transport, and detoxification [55,56,57,58,59] can function with different efficiencies. Metal entry into the aboveground organs depends on the efficiency of transpiration, as it is a driving force for the movement of solutes with mineral elements from roots to shoots. Transpiration rate usually decreases under the influence of Zn [2], Cd [60], Ni [61], and other potentially toxic elements [62]. However, the role of transpiration in metal translocation to the shoots and Zn effects on transpiration rate have not yet been comparatively studied across different populations of *N. caerulescens*. Thus, our study aimed at elucidating whether the intraspecific differences in Zn tolerance and accumulation in the hyperaccumulator *N. caerulescens* are linked to Zn-induced changes in transpiration and mineral composition. To address this issue, for the comparative analyses, we selected two populations of the Zn hyperaccumulator *N. caerulescens*, Prayon and Wilwerwiltz, which contrastingly differ in their habitat, tolerance to Zn, and Zn accumulation capacities [25,45,49,63,64], as well as in population genetics and population biology [65].

## 2. Materials and Methods

### 2.1. Plant Material and Growth Conditions

The Prayon population naturally occurs in a valley that has been contaminated for about 170 years by dust from a Pb–Zn smelter [64,65]. The ammonium acetate-EDTA extractable Zn content in soil there is around 16,000–21,000 mg kg^−1^ [45,63,64]. The Wilwerwiltz population naturally occurs in dry, open grasslands and scrub on shallow, stony non-metalliferous soil containing around 15–36 mg kg^−1^ extractable Zn [45,63,64,65]. Seeds from bulk seed collections from natural populations of the hyperaccumulator *Noccaea caerulescens* F.K. Mey (non-metallicolous population Wilwerwiltz, Luxembourg, 49°59′ N, 05°59′ E, and metallicolous population Prayon, Belgium, 50°34′ N, 05°40′ E) were sown on moist vermiculite. Seed germination and experiments were performed in a climate chamber (20/15 °C day/night; 200 µmol m^−2^ s^−1^ at the plant level, 14 h d^−1^; 70% RH). Two-week-old seedlings were transferred to a hydroponics system, consisting of 5-L PVC pots (28 plants per pot), filled with modified quarter-strength Hoagland’s solution. The pH was set at 5.5 using MES-KOH. When the roots reached 2–3 cm in length, the plants were transferred to 1-L PVC pots (3 plants per pot) with half-strength Hoagland’s solution [25] supplemented with 10 or 500 µM ZnSO_4_. The nutrient solution was replaced once a week. The populations of *N. caerulescens* and Zn concentrations were chosen on the basis of the data on Zn toxic effects and accumulation in different populations of *N. caerulescens* grown hydroponically under similar conditions [21,25,42,46]. The concentration of 10 μM Zn is considered optimal for the growth of *N. caerulescens* [40], whereas 500 μM Zn was shown to be moderately toxic for the root growth of *N. caerulescens* Wilwerwiltz upon short-term exposure [21].

### 2.2. Zinc Effects on Plant Biomass and Water Content

The plants were harvested after 2 months of exposure to Zn. The shoots were excised, and their fresh weight was estimated. The shoots were subsequently washed with demineralized water and blotted dry on filter paper. For metal desorption from the root surface, the roots were sequentially washed with 20 mM Na_2_-EDTA for 10 min at room temperature and then with distilled water. The roots were then blotted dry on filter paper, and their fresh weight was estimated. After that, the plant material was dried in an oven to a constant weight at 95 °C for 72 h and weighed. The root and shoot water contents were calculated as the difference between the fresh and dry weights, expressed as a percentage of the fresh weight. The root-to-shoot dry weight ratio was also calculated.

### 2.3. Zinc Effects on Transpiration Rate

After 2 months of exposure, the plants were put with their roots into 50 mL tubes with the same type of freshly made Zn-amended solution as they were grown on. The plants were then fixed in the holes of the lids by sealing around the stem with Parafilm M (Bemis, Neenah, WI, USA). This approach ensured that evaporation was possible only from the shoot surface. The transpiration rate was measured between 11:00 and 13:00 h as the amount of water evaporated per hour by weighing each plant before and after the treatment as described previously [66], with slight modifications. After the transpiration rate was measured, the shoots were excised, and their fresh weight was estimated. The transpiration rate was calculated per unit shoot fresh weight.

### 2.4. Determination of Zn, Fe, Mn, Cu, Mg, Ca, and K Accumulation in Plants

The dry roots and shoots were powdered, and the samples were digested in 2 mL of a 2:1 mixture of concentrated HNO_3_ (65%) and HClO_4_ (70%) for 5 h at 190 °C. The digests were analyzed for metals, after appropriate dilution with demineralized water (or LaCl_3_ solution, 10 g L^−1^, for Ca quantification), using flame atomic absorption spectrophotometry (AA-7000, Shimadzu, Kyoto, Japan) [21]. To analyze the Zn content in the leaf epidermis and mesophyll, the leaf epidermis was peeled off with tweezers, and the mesophyll was also sampled from at least 6 plants per population (2–3 mg per tissue sample) grown at 500 µM ZnSO_4_. The plant tissue samples were oven-dried, weighed, and processed as described above. The metal translocation factor was calculated as the shoot-to-root metal concentration ratio. “Total metal uptake” was calculated as the sum of the total amount of metal present in the roots and shoots, expressed on a plant dry weight basis. Metal translocation (% translocated) was then recalculated as a percentage of the total metal amount in the shoots relative to the total metal amount taken up by the plant.

### 2.5. Zinc Localization Assay

Zinc localization in the root and leaf tissues was studied after 2 months of exposure to 500 µM Zn. For Zn localization in the root and leaf sections and epidermal peels, we applied Zincon solution [21,67]. Prior to staining, the roots were rinsed with Na_2_-EDTA (20 mM) solution and demineralized water. The sections were examined using an Olympus CX41 microscope (Olympus, Tokyo, Japan). Microphotographs were taken using an MS60 color video camera (Micro-shot Technology Limited, Guangzhou, China). The number of stomata per unit of leaf blade area was calculated on the epidermal peels.

### 2.6. Determination of Photosynthetic Pigments

Chlorophyll *a*, chlorophyll *b*, and carotenoids were extracted in 96% ethanol, and their contents were determined using a standard protocol [68]. The optical density of the alcoholic extracts was measured using a Genesys 180 UV-Vis spectrophotometer (Thermo Fisher Scientific, Waltham, MA, USA) at the wavelengths of 470, 648.6, and 664.1 nm. The pigment concentrations in the extracts were calculated according to the formulas [68] and expressed per unit leaf fresh weight. To visualize leaf chlorosis, microphotographs of the leaf blades were taken using an ADF S645 stereomicroscope (ADF Optics Co., Ltd., Hangzhou, Zhejiang, China) with an ADF Pro 08 digital camera (ADF Optics Co., Ltd., Hangzhou, Zhejiang, China).

### 2.7. Statistical Data Processing

The data were logarithmically transformed and analyzed using two-way ANOVA. A posteriori comparisons of individual means were performed using Tukey’s honest significant difference (HSD) test (*p* ≤ 0.05). Correlations between the metal contents in roots, metal contents in shoots, total uptake of metals, and metal translocation factors, as well as between the percentage of translocated metals, were analyzed using Pearson’s correlation coefficient.

## 3. Results

### 3.1. Zinc Effects on Plant Growth

**Figure 1:**
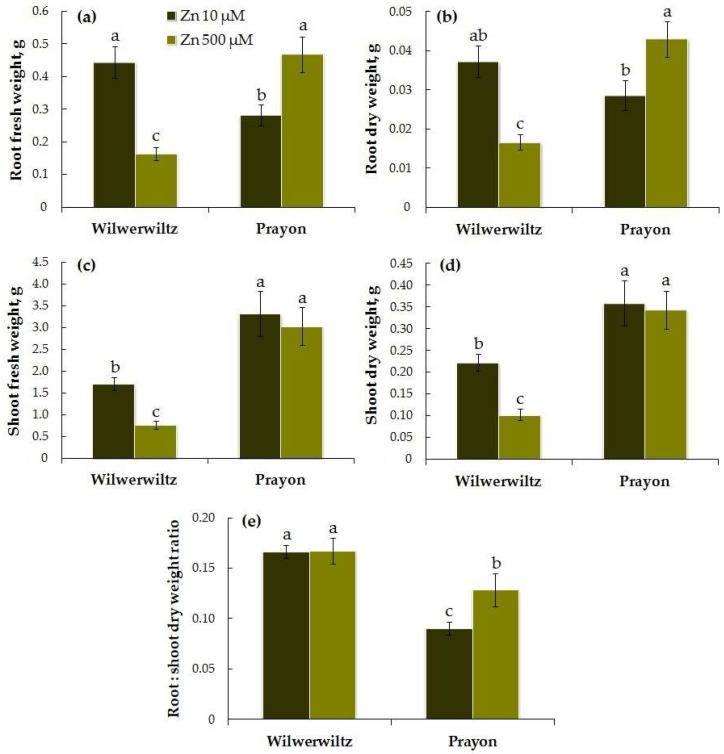
Root fresh (**a**) and dry weights (**b**), shoot fresh (**c**) and dry weights (**d**), and root–shoot dry weight ratio (**e**) after 2 months of Zn treatment of *Noccaea caerulescens* (Wilwerwiltz and Prayon populations) (means ± SEs, n = 9). Values assigned with different letters indicate a significant difference between the means (two-way ANOVA followed by post hoc Tukey’s HSD test, *p* ≤ 0.05).

At 10 µM Zn in the nutrient solution, the root fresh and dry weights of the Wilwerwiltz plants were greater than those of the Prayon plants, although the differences between their root dry weights were not significant. At 500 µM Zn in the medium, the opposite pattern was observed. With the increase in Zn concentration in the solution, the fresh and dry root biomass of the Wilwerwiltz plants significantly decreased, whereas the root biomass of the Prayon plants, in contrast, increased (Figure 1a,b).

At both Zn concentrations in the nutrient solution, the shoot fresh and dry weights were lower in the Wilwerwiltz plants than in the Prayon plants. With the increase in the Zn concentration, a decrease in the shoot fresh and dry weights was observed only in the Wilwerwiltz plants, whereas the shoot fresh and dry weights of the Prayon plants did not change (Figure 1c,d).

The root–shoot dry weight ratio was lower in the Prayon plants than in the Wilwerwiltz plants. An increase in the root–shoot dry weight ratio with increasing Zn concentration in the medium was observed only in the Prayon plants, which was determined by the stimulation of root growth only in this population (Figure 1a,b,e).

### 3.2. Zinc Effects on the Water Content, Contents of Photosynthetic Pigments, and Transpiration Rate

**Figure 2:**
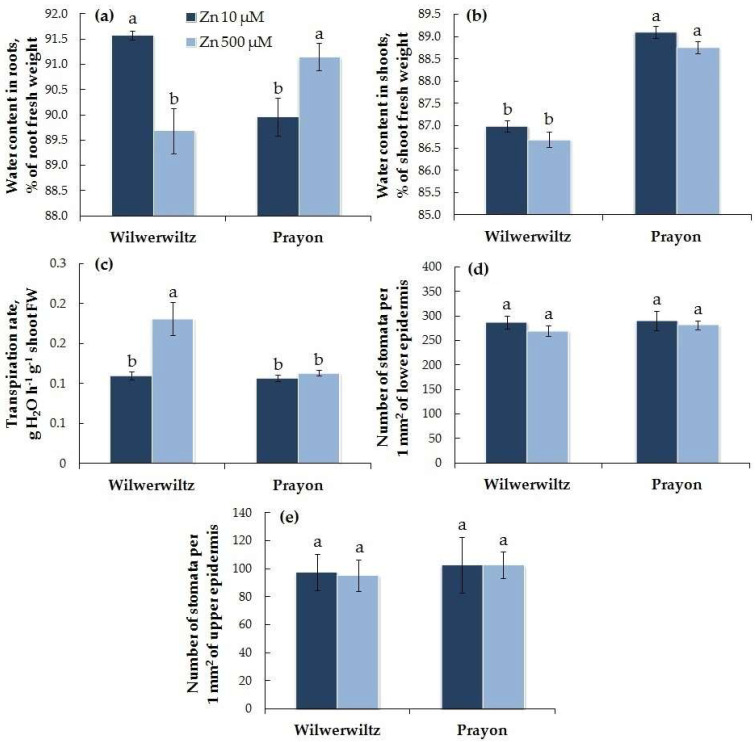
Water contents in roots (**a**) and shoots (**b**), transpiration rate (**c**), and the number of stomata per unit leaf blade area (1 mm^2^) for the lower epidermis (**d**) and upper epidermis (**e**) after 2 months of Zn treatment of *Noccaea caerulescens* (Wilwerwiltz and Prayon populations) (means ± SEs, n = 9). Values assigned with different letters indicate a significant difference between the means (two-way ANOVA followed by post hoc Tukey’s HSD test, *p* ≤ 0.05).

The water content in the roots at 500 µM Zn and in the shoots at both Zn concentrations was lower in the Wilwerwiltz plants than in the Prayon plants, whereas at 10 µM Zn, the water content in the roots was greater in the Wilwerwiltz plants than in the Prayon plants (Figure 2a,b). With the increasing Zn concentration in the medium, the water content decreased in the roots of the Wilwerwiltz plants but increased in the roots of the Prayon plants (Figure 2a), which is consistent with the increase in the root fresh weight in the latter (Figure 1a). The water content in the shoots did not change significantly in either population (Figure 2b).

At 10 µM Zn, the transpiration rate was similar in the plants of both populations, whereas at 500 µM Zn, it was significantly higher in the Wilwerwiltz plants than in the Prayon plants. An increase in the transpiration rate with the Zn concentration in the medium was observed only in the Wilwerwiltz plants, whereas in the Prayon plants, the transpiration rate did not change (Figure 2c). The number of stomata per unit leaf blade area did not change under the Zn treatment in either population (Figure 2d,e).

**Figure 3:**
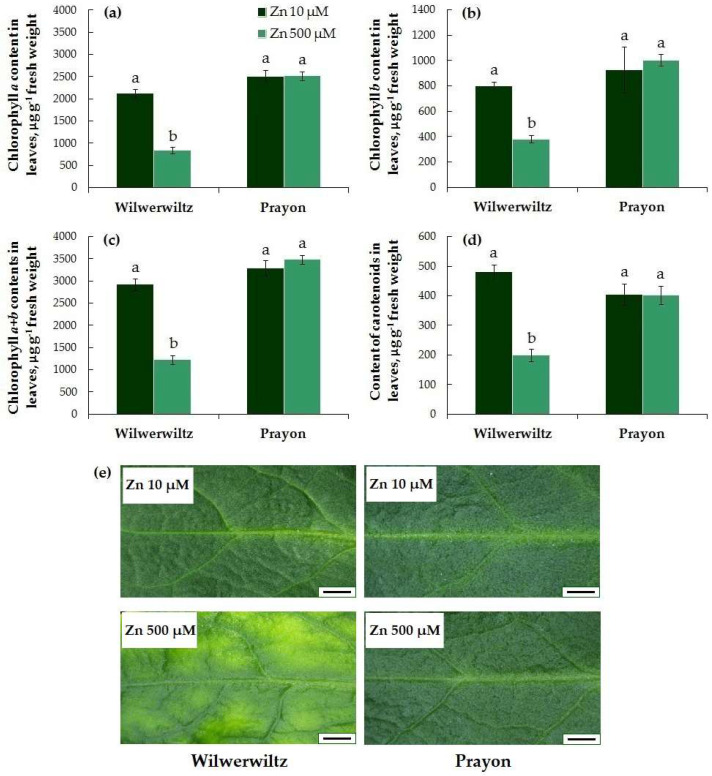
Contents of chlorophyll *a* (**a**), chlorophyll *b* (**b**), chlorophyll *a*+*b* (**c**), and total carotenoids (**d**) in leaves, as well as leaf blade microphotographs (**e**) after 2 months of Zn treatment of *Noccaea caerulescens* (Wilwerwiltz and Prayon populations) (means ± SEs, n = 9). Values assigned with different letters indicate a significant difference between the means (two-way ANOVA followed by post hoc Tukey’s HSD test, *p* ≤ 0.05). Bar, 2 mm (**e**).

Signs of chlorosis were observed only in the Wilwerwiltz plants treated with 500 µM Zn (Figure 3e). This is consistent with the significant decrease in the contents of chlorophylls *a*, *b*, *a*+*b*, and total carotenoids in these plants (Figure 3a–d). In the Prayon plants, no signs of chlorosis or decrease in the contents of photosynthetic pigments were observed (Figure 3).

### 3.3. Accumulation and Distribution of Zn in Roots and Shoots

**Figure 4:**
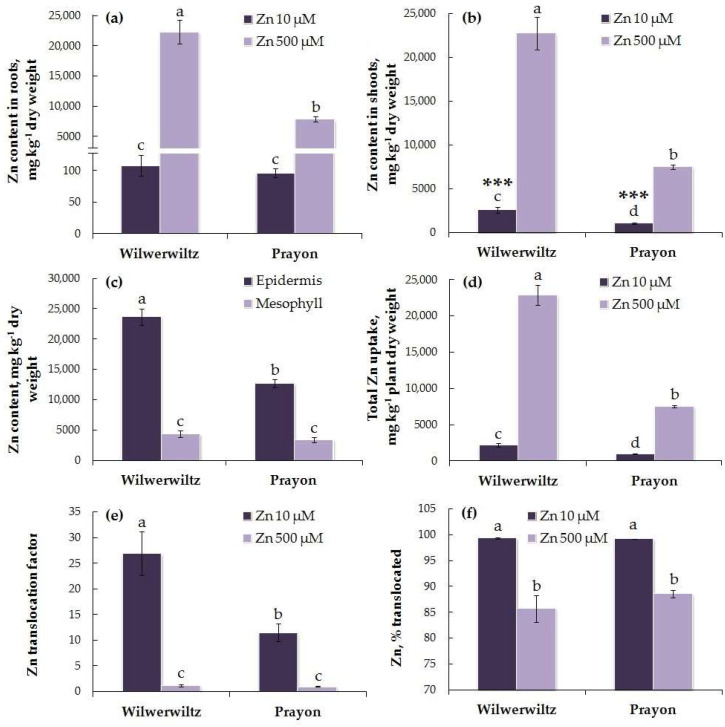
Zinc contents in the roots (**a**), shoots (**b**), leaf epidermis and mesophyll (**c**), total Zn uptake (**d**), Zn translocation factor (**e**), and % translocated (**f**) after 2 months of Zn treatment of *Noccaea caerulescens* (Wilwerwiltz and Prayon populations) (means ± SEs, n = 9). The Zn contents in the leaf epidermis and mesophyll (**c**) were only assessed at 500 µM ZnSO_4_. Values assigned with different letters indicate a significant difference between the means (two-way ANOVA followed by post hoc Tukey’s HSD test, *p* < 0.05). Significant differences between the Zn contents in roots and the Zn contents in shoots are marked by asterisks (one-way ANOVA, *p* ≤ 0.001).

The Zn content in the roots of the Wilwerwiltz and Prayon plants did not differ significantly at 10 µM Zn in the nutrient solution, whereas at 500 µM Zn, it increased by 207-fold in the Wilwerwiltz plants and by 82-fold in the Prayon plants (Figure 4a). At 10 µM Zn, the Zn content in the shoots was higher in the Wilwerwiltz plants than in the Prayon plants. At 500 µM Zn, it increased by 9-fold in the Wilwerwiltz plants and by 7-fold in the Prayon plants (Figure 4b). At the lower Zn concentration in the medium, the Zn content in the shoots was significantly higher than that in the roots, whereas at the higher Zn concentration, the differences between the metal contents in the roots and shoots were insignificant (Figure 4a,b). At 500 µM Zn in the nutrient solution, its content in the leaf epidermis was 5.5 and 3.8 times higher than that in the mesophyll of the Wilwerwiltz and Prayon plants, respectively. The Zn content in the epidermis was significantly higher in the Wilwerwiltz plants than in the Prayon plants, whereas the metal content in the mesophyll was similar (Figure 4c).

The total Zn uptake was greater in the Wilwerwiltz plants than in the Prayon plants at both Zn concentrations. At 500 µM Zn, it increased by 10-fold in Wilwerwiltz and by 8-fold in Prayon (Figure 4d). The Zn translocation factor was >1 in the plants of both populations at both low and high Zn concentrations in the nutrient solution. At 10 µM Zn, the Zn translocation factor was higher in Wilwerwiltz than in Prayon, whereas at 500 µM Zn, no differences were observed between the populations (Figure 4e). Moreover, the percentage of Zn translocated did not differ between the plants of the two populations (Figure 4f). A decrease in the Zn translocation factor and the percentage of Zn translocated was observed in both populations with the increase in the Zn concentration in the medium (Figure 4e,f).

**Figure 5:**
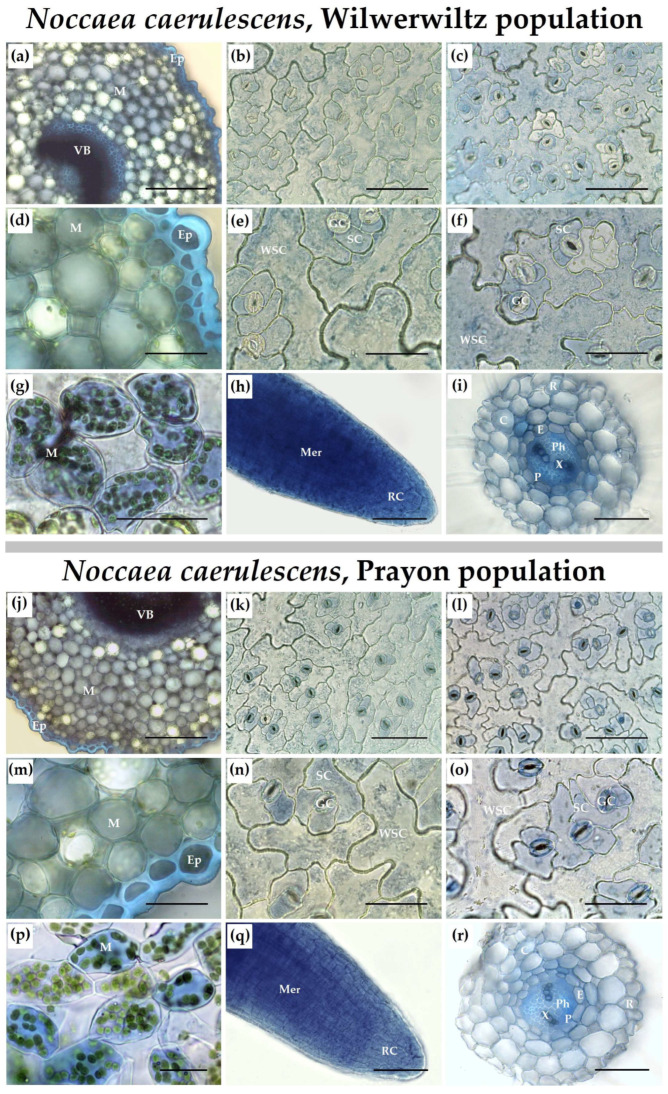
Distribution of Zn over the shoot (**a**–**g**,**j**–**p**) and root (**h**,**i**,**q**,**r**) tissues after 2 months of Zn treatment of *Noccaea caerulescens* (Wilwerwiltz (**a**–**i**) and Prayon (**j**–**r**) populations) with 500 µM Zn. Each picture is a representative selection of six plants per plant type per treatment. (**a**,**d**,**j**,**m**) leaf petiole; (**b**,**e**,**k**,**n**) upper epidermis; (**c**,**f**,**l**,**o**) lower epidermis; (**g**,**p**) leaf mesophyll; (**h**,**q**) root apex; (**i**,**r**) mature root part. C, cortex; E, endodermis; Ep, epidermis; GC, guard cells; M, mesophyll; Mer, meristem; P, pericycle; Ph, phloem; R, rhizodermis; RC, root cap; SC, subsidiary cells; VB, vascular bundle; WSC, water-storage cells; X, xylem. Bars, 50 μm (**d**–**i**,**m**–**r**), 100 μm (**b**,**c**,**k**,**l**), 200 μm (**a**,**j**).

The distribution of Zn over the root and shoot tissues was heterogeneous in the plants of both populations. Zinc was detected both in cell walls and in cell protoplasts. In the roots, Zn accumulated in the meristem (Figure 5h,q), elongation zone, and primordia of the lateral roots. The Zn-dependent staining was more intense in the tissues of the central cylinder than in the rhizodermal or cortical cells (Figure 5i,r). In leaf petioles, Zn was found in the mesophyll cells and accumulated in the vascular bundles and epidermis (Figure 5a,d,j,m). In the leaf blades, Zn was found in the mesophyll (Figure 5g,p) and vascular bundles, whereas in the epidermis, Zn accumulated more in the cells above and below the leaf veins, and its distribution among different cell types was uneven (Figure 5b,c,e,f,k,l,n,o). In the lower (abaxial) leaf epidermis of the Prayon plants, Zn accumulated mainly in the guard and subsidiary cells of the stomatal complex (Figure 5l,o). In the upper (adaxial) leaf epidermis, the differences in Zn accumulation among different cell types were less pronounced compared to the lower epidermis (Figure 5k,n). In the leaves of the Wilwerwiltz plants, the Zn-dependent staining of the water-storage epidermal cells was more pronounced than that observed in the leaves of the Prayon plants, whereas the opposite pattern was observed for the guard cells (Figure 5b,c,e,f,k,l,n,o). The patterns of Zn distribution over the upper and lower leaf epidermis in Wilwerwiltz were similar (Figure 5b,c,e,f). In both populations, the most intense staining of the leaf epidermis was observed above and below the veins.

### 3.4. Effect of Zn on the Contents of Mineral Elements

**Figure 6:**
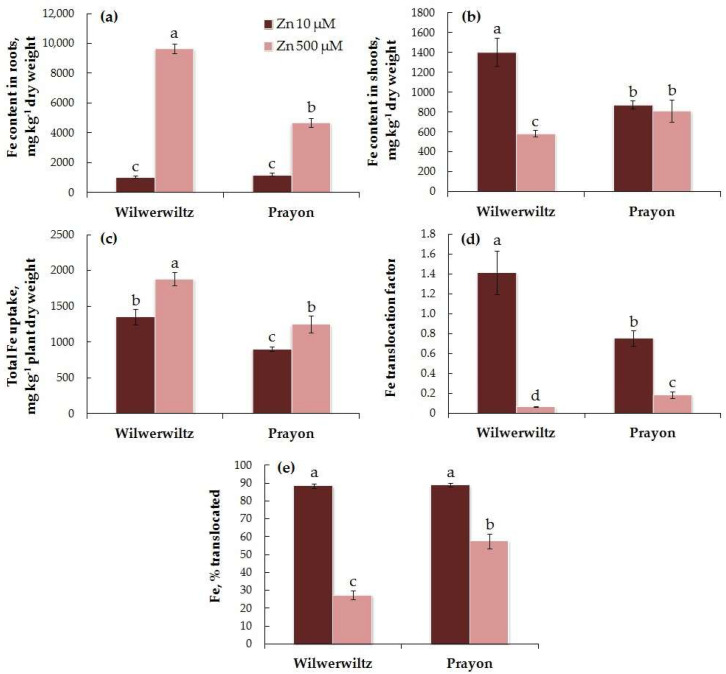
Iron contents in the roots (**a**) and shoots (**b**), total Fe uptake (**c**), Fe translocation factor (**d**), and % translocated (**e**) after 2 months of Zn treatment of *Noccaea caerulescens* (Wilwerwiltz and Prayon populations) (means ± SEs, n = 9). Values assigned with different letters indicate a significant difference between the means (two-way ANOVA followed by post hoc Tukey’s HSD test, *p* ≤ 0.05).

At 10 µM Zn in the nutrient solution, the Fe content in the roots was similar in both populations, whereas at 500 µM Zn, it was higher in Wilwerwiltz than in Prayon (Figure 6a). At 10 µM Zn, the Fe content in the shoots was higher in Wilwerwiltz compared to Prayon, whereas at 500 µM Zn, the opposite pattern was observed (Figure 6b). The Fe content in the roots of both populations increased with the Zn concentration in the medium (Figure 6a, Table 1), which was accompanied by an increase in the total Fe uptake (Figure 6c, Table 1). A significant Zn-induced decrease in the Fe content in shoots was observed only in the Wilwerwiltz population (Figure 6b, Table 1). The percentage of Fe translocated was similar in both populations at 10 µM Zn and was greater in Prayon than in Wilwerwiltz at 500 µM Zn (Figure 6e). A significant decrease in the Fe translocation factor and the percentage of Fe translocated with the increase in the Zn concentration in the medium was observed in both populations and was more pronounced in the plants of the Wilwerwiltz population (Figure 6d,e).

A positive correlation was found between the Zn and Fe contents in the roots, the values of the Zn and Fe translocation factors, the percentage of Zn and Fe translocated, and the total uptake of Zn and Fe in the plants of both populations (Table 2). A negative correlation was found between the Zn and Fe contents in the shoots only in the Wilwerwiltz population (Table 2).

**Figure 7:**
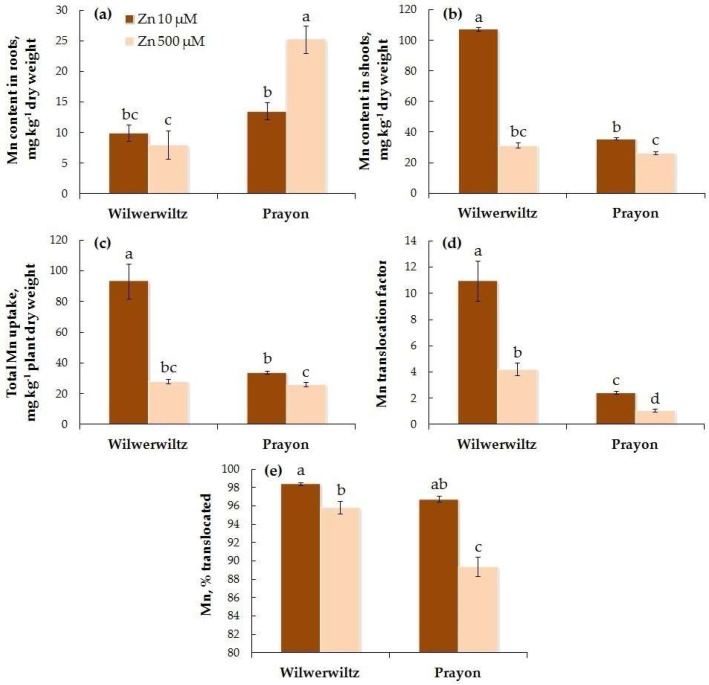
Manganese contents in the roots (**a**) and shoots (**b**), total Mn uptake (**c**), Mn translocation factor (**d**), and % translocated (**e**) after 2 months of Zn treatment of *Noccaea caerulescens* (Wilwerwiltz and Prayon populations) (means ± SEs, n = 9). Values assigned with different letters indicate a significant difference between the means (two-way ANOVA followed by post hoc Tukey’s HSD test, *p* ≤ 0.05).

At 10 µM Zn in the nutrient solution, the Mn content in the roots did not differ between the populations, whereas at 500 µM Zn, it was significantly higher in Prayon than in Wilwerwiltz (Figure 7a). In contrast, the Mn content in the shoots was lower in Prayon than in Wilwerwiltz at the lower Zn concentration, whereas at the higher Zn concentration, it did not significantly differ between the populations (Figure 7b). With the increase in the Zn concentration in the medium, the Mn content in the roots increased in Prayon but did not change in Wilwerwiltz (Figure 7a, Table 1), whereas the Mn content in the shoots, the total Mn uptake, the Mn translocation factor, and the percentage of Mn translocated decreased in both populations (Figure 7b–e, Table 1). In particular, the Mn content in the shoots decreased by 3.5 times in Wilwerwiltz and by 1.4 times in Prayon (Figure 7b, Table 1). The percentage of Mn translocated did not differ between the populations at 10 µM Zn and was greater in Wilwerwiltz than in Prayon at 500 µM Zn (Figure 7e). A positive correlation was found between the Zn and Mn contents in the roots of Prayon, the Zn and Mn translocation factors, and the percentages of Zn and Mn translocated in both populations (Table 2). A negative correlation was found between the Zn and Mn contents in the shoots, as well as the total uptake of Zn and Mn in both populations (Table 2).

**Figure 8:**
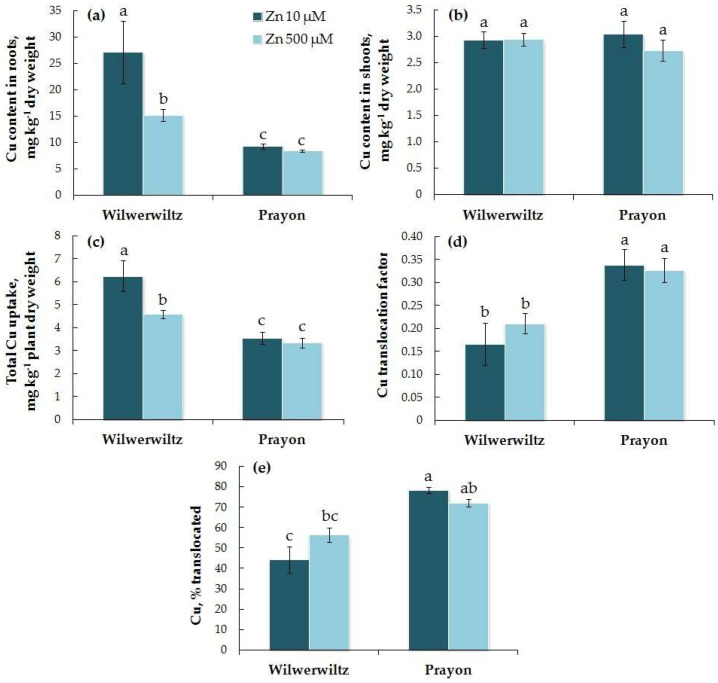
Copper contents in the roots (**a**) and shoots (**b**), total Cu uptake (**c**), Cu translocation factor (**d**), and % translocated (**e**) after 2 months of Zn treatment of *Noccaea caerulescens* (Wilwerwiltz and Prayon populations) (means ± SEs, n = 9). Values assigned with different letters indicate a significant difference between the means (two-way ANOVA followed by post hoc Tukey’s HSD test, *p* ≤ 0.05).

The Cu content in the roots of Wilwerwiltz was higher than that in Prayon, whereas the Cu content in the shoots was similar between the two populations at both Zn concentrations tested (Figure 8a,b). With the increase in the Zn concentration in the medium, the Cu contents in the roots and shoots, the total Cu uptake, the Cu translocation factor, and the percentage of Cu translocated did not change in the Prayon plants, while in the Wilwerwiltz plants, the Cu content in the roots and the total Cu uptake decreased, whereas the Cu content in the shoots, the Cu translocation factor, and the percentage of Cu translocated did not change significantly (Figure 8, Table 1). The percentage of Cu translocated was greater in Prayon than in Wilwerwiltz at 10 µM Zn in the medium. No correlations were found between the Zn and Cu contents in the roots or shoots, nor between the Zn and Cu translocation factors in either population. A negative correlation was found between the total uptake of Zn and Cu in the Wilwerwiltz plants, and a positive correlation was found between the percentage of Zn and Cu translocated in the Prayon plants (Table 2).

**Figure 9:**
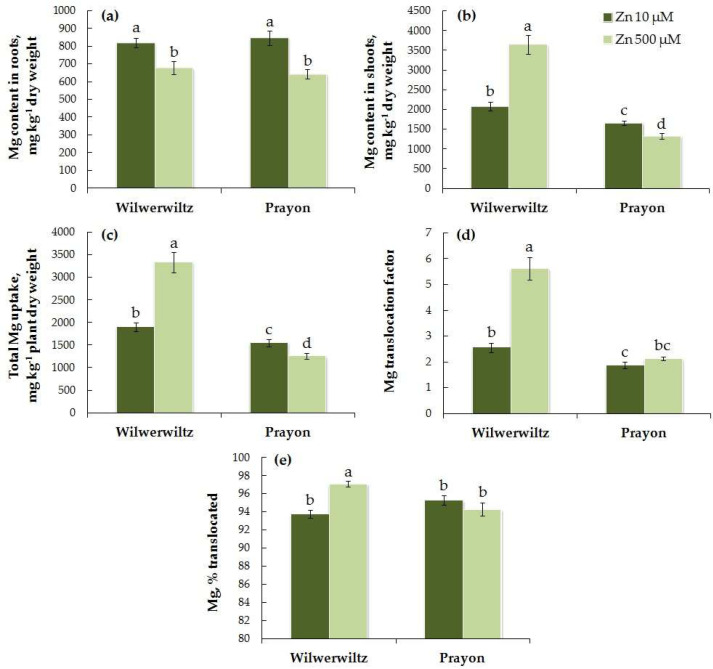
Magnesium contents in the roots (**a**) and shoots (**b**), total Mg uptake (**c**), Mg translocation factor (**d**), and % translocated (**e**) after 2 months of Zn treatment of *Noccaea caerulescens* (Wilwerwiltz and Prayon populations) (means ± SEs, n = 9). Values assigned with different letters indicate a significant difference between the means (two-way ANOVA followed by post hoc Tukey’s HSD test, *p* ≤ 0.05).

The Mg content in the roots did not differ significantly between the populations at either low or high Zn concentrations in the medium, whereas the Mg content in the shoots was higher in Wilwerwiltz than in Prayon (Figure 9a,b). The Mg content in the roots of Wilwerwiltz decreased with increasing Zn concentration in the medium (Figure 9a, Table 1), whereas the Mg content in the shoots increased significantly (Figure 9b, Table 1), which is consistent with the increase in the total Mg uptake, the Mg translocation factor, and the percentage of Mg translocated (Figure 9c–e, Table 1). In Prayon, only a slight but significant decrease in the total Mg uptake, as well as its contents in the roots and shoots, was observed, whereas the Mg translocation factor and the percentage of Mg translocated did not change (Figure 9, Table 1). A positive correlation was found between the Zn and Mg contents in the shoots, as well as between the total uptake of Zn and Mg in the Wilwerwiltz plants (Table 2). A negative correlation was found between the Zn and Mg contents in the roots of both populations, the Zn and Mg contents in the shoots of Prayon, the Zn and Mg translocation factors, and the percentages of Zn and Mg translocated in Wilwerwiltz, and the total uptake of Zn and Mg in Prayon (Table 2).

**Figure 10:**
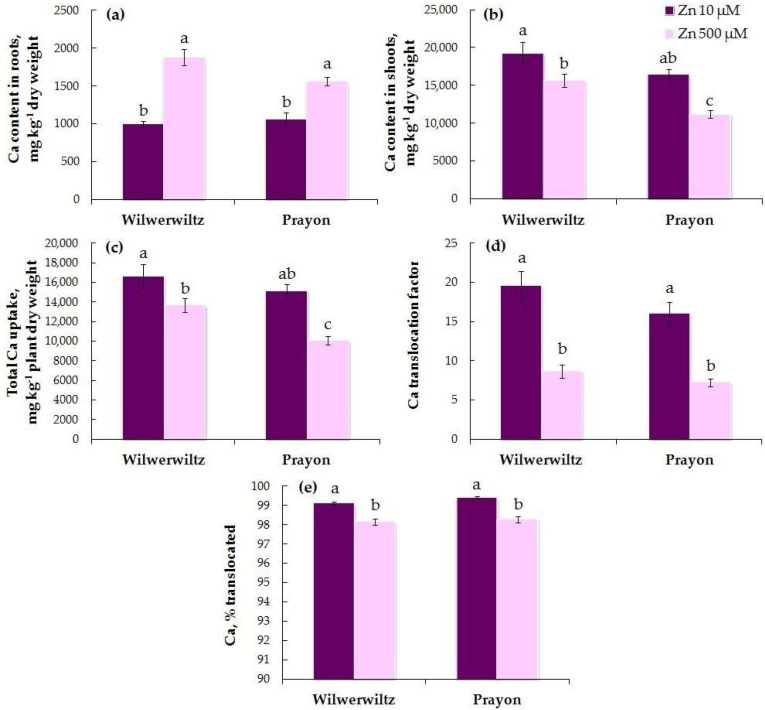
Calcium contents in the roots (**a**) and shoots (**b**), total Ca uptake (**c**), Ca translocation factor (**d**), and % translocated (**e**) after 2 months of Zn treatment of *Noccaea caerulescens* (Wilwerwiltz and Prayon populations) (means ± SEs, n = 9). Values assigned with different letters indicate a significant difference between the means (two-way ANOVA followed by post hoc Tukey’s HSD test, *p* ≤ 0.05).

The Ca content in the roots did not significantly differ between the populations at either Zn concentration in the nutrient solution. The Ca content in the shoots was higher in Wilwerwiltz than in Prayon at 500 µM Zn (Figure 10a,b). With the increase in the Zn concentration in the medium, the Ca content in the roots increased (Figure 10a, Table 1), whereas the Ca content in the shoots decreased (Figure 10b, Table 1) in both populations. This was consistent with a decrease in the total Ca uptake, the Ca translocation factor, and the percentage of Ca translocated (Figure 10c–e, Table 1). A positive correlation was found between the Zn and Ca contents in the roots, the Zn and Ca translocation factors, and the percentages of Zn and Ca translocated in both populations (Table 2). A negative correlation was found between the Zn and Ca contents in the shoots and between the total uptake of Zn and Ca in the Prayon plants (Table 2).

**Figure 11:**
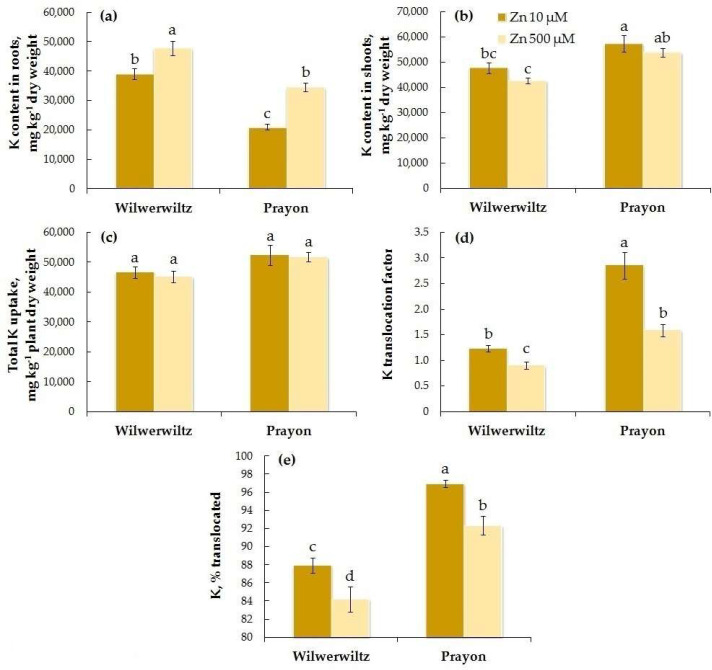
Potassium contents in the roots (**a**) and shoots (**b**), total K uptake (**c**), K translocation factor (**d**), and % translocated (**e**) after 2 months of Zn treatment of *Noccaea caerulescens* (Wilwerwiltz and Prayon populations) (means ± SEs, n = 9). Values assigned with different letters indicate a significant difference between the means (two-way ANOVA followed by post hoc Tukey’s HSD test, *p* ≤ 0.05).

The K content in the roots was higher in Wilwerwiltz than in Prayon at both Zn concentrations in the nutrient solution, whereas the opposite pattern was observed for the K content in the shoots (Figure 11a,b). With the increase in the Zn concentration in the medium, the K content in the roots increased in both populations (Figure 11a, Table 1), whereas its content in the shoots slightly, although insignificantly, decreased (Figure 11b, Table 1), which is consistent with the decreases in the K translocation factor and the percentage of K translocated (Figure 11d–e, Table 1). Total K uptake did not change with the increase in the Zn concentration in either population (Figure 11c, Table 1). A positive correlation was found between the Zn and K contents in the roots, Zn and K translocation factors, and the percentages of Zn and K translocated in both populations (Table 2).

A positive correlation was also observed between the contents of Fe and Ca, Fe and K, Ca and K in the roots in both populations; the contents of Fe and Mn, Cu and Mg, Mn and Ca, Mn and K in the roots of Prayon; the contents of Fe and Mn, Fe and Ca, Fe and K in the shoots of Wilwerwiltz; the contents of Mn and Mg, Mn and Ca in the shoots of Prayon; the Fe and Mn, Fe and Ca, Fe and K, Mn and Ca, K and Ca translocation factors and percentages translocated in both populations; the Mn and K translocation factors and percentages translocated in Prayon; the percentages of translocated Cu and Mn, Cu and Mg, Cu and Ca, Cu and K, Mg and Ca, Mg and K in Prayon; as well as the total uptake of Fe and Mg in Wilwerwiltz, and that of Mn and Mg, Mn and Ca in Prayon (Table 2). A negative correlation was found between the Fe and Mg contents in the roots of both populations; between the K and Mg contents in the roots of Prayon; the Mn and Ca, Mg and Ca, Cu and K contents in the roots of Wilwerwiltz; the Fe and Mg, Mg and Mn contents in the shoots of Wilwerwiltz; the Mg and Fe, Mg and Mn, Mg and Ca translocation factors and percentages translocated in Wilwerwiltz; the Mg and Mn translocation factors in Prayon; the Mg and K translocation factors in Wilwerwiltz; as well as the total uptake of Fe and Mn, Fe and Cu, Mg and Mn, Mg and Cu in Wilwerwiltz, and that of Mg and Fe in Prayon (Table 2).

## 4. Discussion

### 4.1. Plant Tolerance to Zn

The symptoms of toxicity are manifested at different metal contents in plants of different species and populations, which reflects the degree of their metal tolerance and metal accumulation capacity [25,41,42,46]. Root growth is significantly more tolerant to Zn in hyperaccumulators compared to excluders [21,25]. However, Zn tolerance can vary greatly among plants from different populations of the same hyperaccumulator species. In the Wilwerwiltz population grown at 500 µM Zn, the metal toxic effects included a decrease in the accumulation of fresh and dry root and shoot biomass (Figure 1), water content in the roots (Figure 2), and the contents of photosynthetic pigments in the shoots, which was accompanied by the appearance of leaf chlorosis (Figure 3). A decrease in the water content in plants is a phenomenon often observed under the action of various metals, including Zn [15,21,69,70], and metal-induced loss of turgor and disruption of cell elongation eventually result in growth inhibition and a decrease in biomass.

In contrast to Wilwerwiltz, no signs of metal toxicity were observed in the Prayon population at 500 µM Zn (Figure 1, Figure 2 and Figure 3), which may be partly due to its evolution on Zn-enriched calamine soils and to the lower Zn content in the Prayon plants than in the Wilwerwiltz plants (Figure 4a,b). A comparative analysis of 28 populations of *N. caerulescens* revealed that root growth, depending on the population, was completely inhibited by 2750–5750 µM Zn in the nutrient solution. The populations of *N. caerulescens* from calamine soils were significantly more tolerant to Zn than the populations from non-metalliferous and serpentine soils. The populations from non-metalliferous soils were more tolerant to Zn than those from serpentine soils. The lethal concentration of Zn in the nutrient solution estimated by the root test was 5000 µM for the Prayon population from the calamine soils of Belgium and 4250 µM for the Wilwerwiltz population from the non-metalliferous soils of Luxembourg [25], which is consistent with the greater tolerance of Prayon compared to Wilwerwiltz, as assessed by various parameters (Figure 1, Figure 2 and Figure 3). Among the populations tested, the most tolerant was the Lanestosa population, originating from Zn-enriched calamine soils of Spain, in which complete cessation of root growth was observed at 5750 µM Zn [25]. Moreover, even *N. caerulescens* populations from non-metalliferous soils were much more tolerant to Zn than were the closely related excluders *Thlaspi arvense* and *Microthlaspi perfoliatum*, in which complete cessation of root growth was detected at 250 μM Zn in the nutrient solution [25]. The high Zn tolerance of the populations from non-metalliferous soils suggests the possibility that the capacity to hyperaccumulate Zn emerged not only on metalliferous soils, but also on non-metalliferous soils, followed by colonization of metalliferous soils and an increase in plant metal tolerance [25,38].

When the Zn concentration in the nutrient solution was increased from 10 to 500 µM, a stimulation of root growth, but not shoot growth, was observed in the Prayon population (Figure 1a,b). In the presence of moderate Zn concentrations, the hyperaccumulators *N. caerulescens* and *Arabidopsis halleri* developed better than the control plants did [46,67,71]. Interestingly, the Prayon population of *N. caerulescens* exhibited zincophilic root foraging patterns in response to heterogeneously distributed Zn, suggesting that it can distinguish between patches with different Zn concentrations and produce more roots in the patches with higher Zn concentrations [72]. The Zn concentrations at which the stimulation of root growth is observed are significantly higher for the hyperaccumulators than for the excluders and can vary depending on the population. For example, the Zn concentrations optimal for the growth of the St-Félix-de-Palliéres and St-Laurent-le-Minier (Ganges) populations of *N. caerulescens* from calamine soils in France were 100–400 and 100–200 µM Zn, respectively [46,67]. However, short-term exposure of Wilwerwiltz plants to 5–500 µM Zn did not stimulate root growth [21]. The Zn-hyperaccumulating ecotype of *Sedum alfredii* grew better at high Zn levels in shoots (29.11 g kg^−1^ dry weight) [20], and the root length, root surface area, and root volume were significantly increased even under 500 µM of Zn [73], which is consistent with our data for the Prayon population of *N. caerulescens* (Figure 1a,b). Higher concentrations of Zn that are optimal for the growth of hyperaccumulators indicate their higher tolerance to Zn due to effective mechanisms of its detoxification [36,50,53,74,75] and, possibly, a greater requirement for Zn in these species in order to protect themselves against herbivores and pathogens [76,77].

### 4.2. Zn Accumulation in Roots and Shoots

The ability of *N. caerulescens* to accumulate metals depends not only on the growth conditions and metal concentration in the medium, but also on the population under study and the duration of exposure [21,25,40,41,42,45,46,78], which makes it difficult to compare the data obtained in different works. During the short-term treatments (3–9 days) of hydroponically grown Wilwerwiltz plants with 2000–4000 µM Zn, the Zn content in the shoots was lower than that in the roots, which is consistent with the translocation factor values below 1 [21]. During the long-term exposure (2 months) to 10 µM Zn, the Zn content in the shoots of plants of this population exceeded its content in the roots (Figure 4a,b), and the average translocation factor value reached 27 (Figure 4e). In the Prayon plants, the average value of the Zn translocation factor at 10 µM Zn was significantly lower, reaching 11.5 (Figure 4e). At 5 µM Zn in the nutrient solution, among the 19 populations under study, the highest translocation factor (5.83) was shown for the Prayon population and did not differ significantly from that for the Wilwerwiltz population, whereas the lowest translocation factor (0.37) was observed for the plants of the Les Avignières population from the calamine group [42]. At 500 µM Zn, the translocation factor decreased in Wilwerwiltz and Prayon due to greater Zn accumulation in the roots, but still exceeded 1 (Figure 4e), which is typical of hyperaccumulators. Importantly, the percentage of Zn translocated did not differ between the two studied populations at either low or high Zn concentration, and decreased equally with the increase in the Zn concentration in the nutrient solution (Figure 4f). Thus, greater Zn accumulation in the shoots of Wilwerwiltz plants was due to higher Zn uptake (Figure 4b,d). Taken together, the data obtained not only indicate significant differences in the ability of plants to accumulate Zn among the *N. caerulescens* populations [25,42], but also point to changes in the intensity of Zn root-to-shoot translocation depending on the metal concentration and duration of exposure. Similar patterns were previously shown by us for different populations of *N. caerulescens* grown hydroponically in the presence of Ni [25,46].

Metal accumulation in plants grown hydroponically and in soil can differ significantly, which does not allow direct comparison of these data, even at similar metal contents in the environment. Indeed, the plants of several populations of *N. caerulescens* from calamine and non-metalliferous soils grown hydroponically accumulated similar amounts of Zn, but the metal content in them was three times higher than that in the plants of the same populations grown in soil [45]. This may be due to the heterogeneity of the soil, uneven metal distribution across the soil profile, and different ratios of macro- and microelements. Interestingly, the ratio of shoot Zn content in plants of the non-metallicolous population Wilwerwiltz to that in plants of the metallicolous population Prayon varied strongly with the form of Zn in the soil [49]. In a comparative study of 28 populations of *N. caerulescens* grown hydroponically at 5 µM Zn, the highest Zn content in shoots was observed in the calamine populations Pontaut from Spain and Col du Mas de l’Ayre from France [25], whereas the highest Zn content in roots was found in the calamine population Les Avinières from France. No correlation was found between the Zn content in the roots and the Zn content in the shoots of plants from different populations of *N. caerulescens* [42]. However, at 500 µM Zn in the nutrient solution, the Zn contents in the roots and shoots of the Wilwerwiltz plants were several times higher than those in the Prayon plants (Figure 4a,b). A similar pattern was observed when the plants of these populations were grown in soil spiked with 2000 mg kg^−1^ Zn, which allowed Wilwerwiltz to be considered a better phytoextractor of Zn for use in phytoremediation, except in substrates with low pH and high concentrations of free Zn in the soil solution [49]. The Zn content in the leaves of the hydroponically grown plants of the non-metallicolous population Lellingen was also greater compared to that in the calamine populations La Calamine and St. Fèlix de Palliéres [46]. In general, non-metallicolous populations were found to accumulate more Zn than metallicolous populations when grown in moderately Zn-enriched soil [41,45,49,63]. Therefore, it has been suggested that the Zn accumulation capacity may be genetically predetermined [41].

In the natural habitat, in soils with <50 mg kg^−1^ extractable Zn and approximately 140 mg kg^−1^ total Zn, a population from Luxembourg accumulated ~3230–8890 mg Zn kg^−1^ dry weight in leaves [48]. Such a high level of Zn accumulation might be achieved, at least to some extent, through the continuous proliferation of lateral roots in the areas enriched with Zn, thus reallocating root growth towards soil areas not yet depleted of readily available Zn [72,79,80]. The toxic effect of Zn on Prayon plants was not observed even when its content in roots and shoots exceeded 7000 mg kg^−1^ dry weight (Figure 4a,b). The absence of a decrease in biomass accumulation at similar Zn contents in plants was also observed for several other populations of *N. caerulescens* [46] and for the Zn hyperaccumulator *Arabis paniculata*, which accumulated up to 12,892 mg Zn kg^−1^ dry weight in roots and up to 6030 mg Zn kg^−1^ dry weight in shoots [81]. Interestingly, at a similar degree of root growth inhibition (~70%) observed on the sixth day of exposure, the Zn content in the roots of *N. caerulescens* (Wilwerwiltz population) reached ~12,300 mg kg^−1^ dry weight in plants treated with 3000 µM Zn, whereas in the roots of the non-accumulator *M. perfoliatum* treated with 20 µM Zn, it reached ~2400 mg kg^−1^ dry weight, which was approximately five times lower [21]. These differences may be associated with more effective Zn detoxification mechanisms in hyperaccumulators than in excluders [36,50,53,74,75]. At the same time, a significant negative correlation between the Zn content in the roots of plants of different populations of *N. caerulescens* and their Zn tolerance, as assessed by the root test, indicates a clear dependence of the degree of root growth inhibition on the amount of metal accumulated in the roots [42].

### 4.3. Zinc Distribution over the Root and Shoot Tissues

An uneven Zn distribution in the root and shoot tissues was observed in both populations (Figure 5). In the roots, Zn was accumulated in the meristem (Figure 5h,q), indicating the absence of any physiological barriers to its uptake in the apical part of the root. This may be determined by both the structural features of the cell walls of meristematic cells [82] and the capacity for Zn translocation along the symplast in the root meristem, as evidenced by its localization in the protoplasts of the meristematic cells (Figure 5h,q). The accumulation of Zn in root meristematic cells was shown for *Capsella bursa-pastoris*, *Lepidium ruderale* [83], *M. perfoliatum* [21], *Triticum aestivum* [84], and *Zea mays* [85]. In *N. caerulescens*, the accumulation of Zn in the apical part of the root was observed already after 3 days of treatment [21]. As in the growing part of the root, the Zn content in the lateral root primordia was significantly higher than that in the other parts of the root, which was also observed in the excluders [83,85]. The accumulation of Zn in the growing part of the root can be directly related to Zn requirement for growth processes [4,21]. At the same time, the accumulation of Zn in the meristem and elongation zone is one of the reasons for its growth-inhibitory effect [21,85]. The toxic effect of Zn on cell division and elongation in *N. caerulescens* is manifested, however, at a significantly higher Zn concentration in the medium and its endogenous content in planta than in the closely related excluder *M. perfoliatum* [21], which is due to more effective Zn detoxification mechanisms functioning in the hyperaccumulator [36,50,53,74,75].

Quantitative and histochemical analyses revealed that the Zn content in the leaf epidermis was much higher than that in the mesophyll in both populations (Figure 4c and Figure 5a–g,j–p). Similar results were obtained using micro-PIXE analysis of hydroponically grown plants of the Prayon population, in which, at 300 µM Zn in the nutrient solution, Zn was preferentially distributed in the leaf epidermis and was detected in the mesophyll to a relatively smaller extent [86]. Synchrotron-based micro-X-ray fluorescence (μXRF) analysis of Zn localization in the leaves of *Noccaea praecox* also showed the strongest enrichment in the epidermal cells, whereas the Zn accumulation in the vascular bundles and mesophyll was less pronounced [87]. Notably, a high enrichment in Zn was detected around the central and secondary veins in *N. praecox* [87], as well as in *N. caerulescens* using synchrotron-based X-ray fluorescence analysis [88] and histochemical analysis, particularly in the epidermis above/below the veins (present study and [67]). The accumulation of Zn in the leaf epidermis serves as an effective mechanism for its detoxification, aimed at reducing Zn entry into the mesophyll and its effect on photosynthesis [53]. The proportion of Zn accumulated in the epidermis and mesophyll can vary depending on the plant anatomical and physiological characteristics. In the leaves of *A. halleri*, Zn was detected mainly in the mesophyll, as well as at the base of trichomes [71,89], whereas in *S. alfredi* [90], *N. praecox* [91], and *N. caerulescens* [67,86,92], Zn accumulated mainly in the epidermis, which is consistent with the data obtained in the current study. Such differences can be partly explained by the larger size of epidermal cells in the representatives of the genus *Noccaea* than those in *A. halleri* [71,89]. However, since the mesophyll occupies a greater fraction of the leaf volume than the epidermis does, the total Zn content in the mesophyll of *N. caerulescens* can be significant and even exceed the total metal content in the epidermis [92].

Zinc accumulation in leaf epidermal cells can be associated with both an increased number of transporters belonging to the ZIP family, which are responsible for Zn transport across the plasma membrane in these cells [93], and the “passive accumulation” of Zn due to its transport along with the transpiration-driven water stream, which ends in the epidermis [94]. Transpiration provides the driving force for the root-to-shoot transport of water and mineral elements via the xylem. In addition, the ratio of water uptake rate to transpiration rate determines the maintenance of water balance in plants. Typically, the toxic effect of Zn is manifested as a decrease in the transpiration rate, which serves to avoid the disruption of water balance as a result of Zn accumulation in the shoots [2]. However, at 500 µM Zn, no changes in shoot water content were detected in the plants of either population (Figure 2b), and the transpiration rate of the Wilwerwiltz plants increased compared to that of the control (Figure 2c). The increase in the transpiration rate was not associated with the changes in the stomatal density, since the latter was not affected by the Zn treatment (Figure 2d,e). Thus, the higher Zn content in the leaf epidermis of Wilwerwiltz compared to that of Prayon (Figure 4c) may be partly due to the higher transpiration rate in the former, at least at 500 µM Zn (Figure 2c).

Zinc was unevenly distributed among different types of leaf epidermal cells, and the pattern of its distribution differed between the Wilwerwiltz and Prayon plants. In Prayon, Zn accumulated mainly in the guard and subsidiary cells of the stomatal complex (Figure 5k,l,n,o), whereas in Wilwerwiltz, it accumulated in the large water-storage (also called “metal-storage”) pavement cells of the epidermis (Figure 5b,c,e,f). The accumulation of Zn in the water-storage cells is consistent with the higher Zn content in the leaf epidermis of Wilwerwiltz compared to that of Prayon (Figure 4c). Zn accumulation in the water-storage cells of the leaf epidermis was also shown for *N. praecox* [91] and different populations of *N. caerulescens* [67,95]. In plants of the St-Laurent-le-Minier (Ganges) population of *N. caerulescens*, which grew naturally at a mine in Southern France as well as those grown in the laboratory, Zn accumulated to a greater degree in the stomatal guard cells and water-storage epidermal cells than in the subsidiary cells of the stomatal complex [67]. The uneven distribution of Zn among different types of leaf epidermal cells may be partly determined by the heterogeneity of the distribution of transporters, which are located at the plasma membrane and provide Zn entry into the cytosol. In young leaves of young plants of the St-Laurent-le-Minier (Ganges) population of *N. caerulescens*, the level of *NcZNT5* mRNA in the large metal-storage epidermal cells was significantly greater than that in the subsidiary and guard cells, whereas in mature leaves, the level of *NcZNT1* expression was greater in the guard cells than in other types of epidermal cells [96]. Since Zn accumulation in different types of epidermal cells may vary with leaf age, it is important to note that in the present work, mature leaves of the same age from plants of the two populations were studied. Therefore, the existence of intraspecific differences in the localization of transporters, which are indicated by the differences in the Zn distribution in the two populations, also cannot be ruled out.

In the leaf blades, Zn accumulation was detected in the protoplasts of epidermal cells (Figure 5b,c,e,f,k,l,n,o). In a mature plant cell, up to 90% of the cell volume may be taken up by the vacuole. Therefore, it may be assumed that a significant part of the Zn entering the cell accumulates in the vacuole, which was confirmed for the Prayon population by the data obtained from ultrathin cryosections using energy-dispersive X-ray micro-analysis [95]. The vacuolar storage of Zn was also confirmed when the Zn fluorescent indicator Newport Green was used to visualize Zn within the protoplasts isolated from the leaf cells of *A. halleri* [97]. Metals, including Zn, enter the vacuole via various transporters, such as the tonoplast Zn^2+^/H^+^ antiporter MTP1 and the P_1B_-type ATPase HMA3 [51,53,98,99]. In young and mature leaves of *N. caerulescens*, very high levels of *MTP1* transcripts were detected in the water-storage epidermal cells [96]. The level of *NcHMA3* transcripts was seven times higher in the plants of the St-Laurent-le-Minier (Ganges) population of *N. caerulescens* than that in the plants of the Prayon population, which was partly caused by a higher gene copy number, although NcHMA3 was more specific for Cd than for Zn [55]. The accumulation of Zn in the vacuoles, where it is bound to various ligands, mainly organic acids [53,75,100], is one of the most important mechanisms of Zn detoxification.

The uneven distribution and accumulation of Zn in plant tissues can be determined not only by the heterogeneity of the distribution of the corresponding transporters [96,101] but also by the different contents of low-molecular-weight ligands in the cells of different tissues [93]. Using a proteomic approach, it was shown that almost all of the Zn located in the mesophyll of *N. caerulescens* was stored as complexes with the non-proteinogenic amino acid nicotianamine, whereas in the epidermis, the proportion of these complexes was significantly lower, and Zn was associated mainly with citric and malic acids [93]. Due to the uneven distribution of metals, their toxic effects in different tissues and even in different cells of the same tissue can be manifested differently [102].

Considering that a larger fraction of a leaf volume is occupied by the mesophyll than by the epidermis, it becomes obvious that when metal accumulation in the epidermis exceeds a certain critical value, its contents in the mesophyll can increase significantly. In the mesophyll, metals can bind to the cell wall material, be sequestered in the vacuole, and can also enter other organelles, including chloroplasts and mitochondria, where, at elevated concentrations, they exert multiple toxic effects on photosynthesis and respiration [2,53,60,61]. It is possible that the P_1B_-type ATPase HMA4 is involved in Zn translocation from the mesophyll to the epidermis [93], whereas MTP1, which is involved in the Zn entry into the vacuole, was found not only in the epidermis but also in the mesophyll [93,96]. At 500 µM Zn in the medium, Zn content in the mesophyll of Wilwerwiltz and Prayon leaves did not differ significantly (Figure 4c). Therefore, the manifestation of the toxic effect of Zn in the shoots of only Wilwerwiltz is not associated with a more intensive metal entry into the mesophyll cells in Wilwerwiltz than in Prayon. It can be assumed that the latter has more effective mechanisms of Zn detoxification in the cells due to its evolution on calamine soils.

### 4.4. Zn Uptake, Transport, and Detoxification

Differences in Zn accumulation capacity between *N. caerulescens* populations may be related to differences in the efficiency of Zn uptake and root-to-shoot translocation. The total Zn uptake of the Wilwerwiltz plants was greater than that of the Prayon plants and increased with the Zn concentration in the nutrient solution (Figure 4d). At 2000–4000 µM Zn, total Zn uptake increased in the Wilwerwiltz plants with the increase in the duration of treatment from 3 to 9 days [21]. The identified differences between the populations were similar when the plants were grown both in soil and in hydroponics. Compared with the plants of the calamine population Prayon, the non-metallicolous population Wilwerwiltz had constitutively greater Zn uptake capacity and ability to obtain Zn from specific Zn salts [49]. Assunção et al. [103] compared the Zn uptake capacity in hydroponically grown metallicolous and non-metallicolous populations of *N. caerulescens* and reported a greater accumulation capacity in the non-metallicolous population despite the absence of enhanced transcription of the *ZNT1* and *ZNT2* genes in that population. However, the mechanisms of Zn, Ni, and Cd uptake can differ significantly among the populations of *N. caerulescens* [54]. In the Lellingen, St-Laurent-le-Minier (Ganges), Monte Prinzera, and La Calamine populations, a highly Zn-preferential high-affinity system for Zn uptake was found, whereas a system with a relatively low affinity for Zn, compared to Ni and Cd, was shown only in the Lellingen population from non-metalliferous soil and the St-Laurent-le-Minier (Ganges) population from calamine soil, which partly explains the low rate of metal accumulation by La Calamine plants, both under natural growth conditions on Belgian calamine soil [54] and in hydroponics [46,104]. A significant loss of Zn hyperaccumulation capacity in some calamine populations from the Eifel region, including La Calamine [25,104], results from a loss of functional IRT1 [105], a poorly metal-specific iron transporter involved in the transport of divalent Fe, Zn, Ni, Co, Mn and Cd ions into the cytosol [53,106,107,108]. IRT1 is believed to contribute substantially to intraspecific variation in Zn/Cd or Ni hyperaccumulation capacities among populations of several metallophyte species [3,52,105].

Several mechanisms may be involved in the higher Zn accumulation in non-metallicolous populations, including a higher root–shoot ratio, greater specific root length, and enhanced Zn root-to-shoot translocation [49]. In general, the plants from the calamine group of populations differ in a number of morphological features from the plants of non-metallicolous and serpentine groups. They have a greater rosette diameter, shoot biomass, and number of inflorescences [41]. At 500 µM, the biomass of roots and shoots was greater in Prayon than in Wilwerwiltz, which is associated with, among other factors, the stimulation of root growth in Prayon (Figure 1a–d). Wilwerwiltz had a greater root–shoot ratio compared to Prayon (Figure 1e). Therefore, one of the explanations for the lower metal hyperaccumulation in the metallicolous population might be a mere “dilution effect” due to the higher biomass in this population, although this factor is not decisive [64].

In cells, the concentration of “free Zn” or “labile Zn”, which refers to the Zn^2+^ ions not tightly bound to proteins, is rather low [109]. For an average-sized root cell, the concentration of 420 pM would yield approximately 100 free (or loosely bound) Zn^2+^ ions per cell cytosol, while millions of Zn^2+^ ions would be bound to proteins [110] and low-molecular-weight ligands such as nicotianamine and histidine [53,75]. Therefore, one of the reasons for the metal hypertolerance of hyperaccumulators may be the high level of intracellular metal-binding ligands in the roots, which ensures efficient binding of metal ions in the cytosol, facilitates metal radial transport along the symplast, loading into the xylem vessels, and entry into the shoots [53,109,111,112]. Binding of Zn to low-molecular-weight ligands in the cytosol limits its entry into the vacuoles of root cortical cells in *N. caerulescens* [53,113], which is consistent with its localization mainly in the tissues of the central cylinder in mature root parts (Figure 5i,r), as well as with the data on Zn quantification in the root vacuoles [114]. However, hydroponically grown populations of *N. caerulescens*, which differ significantly in their tolerance and ability to accumulate Zn, Ni, and Cd [25,42,46,104,115], presented similar free histidine levels in roots and shoots [115]. Therefore, intraspecific differences in metal tolerance and metal accumulation capacities, at least in *N. caerulescens*, are not determined solely by the increased endogenous level of free histidine in the roots. Moreover, plants of different populations of *N. caerulescens* may differ in the level of expression of *NAS* genes encoding the key enzyme of nicotianamine biosynthesis, nicotianamine synthase, which may partly determine their different capacities to accumulate metals [57,59].

Histochemical analysis revealed that Zn is present not only in cell protoplasts, but also in cell walls (Figure 5), which was also shown for different populations of *N. caerulescens* [21,95,113], as well as for other species [83,85,113,114,116]. Metal symplastic mobility in hyperaccumulators can be facilitated by their entry from the apoplast, which is mediated by the NRAMP1 transporter located at the plasma membrane of the endodermal and stele root cells in *N. caerulescens*. In the roots of the St-Laurent-le-Minier (Ganges) plants, the expression level of *NcNramp1* was at least five times higher than that in the Prayon plants, which resulted in a more efficient passage of the endodermal barrier and further xylem loading and translocation of Cd to the shoots in the former [58]. Since this protein can transport not only Cd but also Zn in the roots and shoots of *S. alfredii* [117], the functioning of a similar mechanism in the case of Zn in *N. caerulescens* cannot be excluded.

Intraspecific differences in Zn accumulation can be determined at the level of its loading into the xylem vessels, the efficiency of which can vary significantly among different populations of *N. caerulescens* [104]. Symplastic chelators, such as histidine and nicotianamine, may deliver metals to the proteins involved in metal loading into the xylem, for example, Zn to the P_1B_-type ATPases HMA2 or HMA4 [53,75]. The expression level of the *NcHMA4* gene in the Puente Basadre population of *N. caerulescens* originating from ultramafic soils in Spain was lower than that in the Prayon, St-Laurent-le-Minier (Ganges), and St-Félix-de-Palliéres populations originating from calamine soils. This is associated with the different numbers of the *NcHMA4* gene copies: four in the St-Laurent-le-Minier (Ganges) and St-Félix-de-Palliéres populations, three in the Prayon population, and just two in the Puente Basadre population [56]. However, the Zn accumulation capacity of different *N. caerulescens* populations may differ despite similar levels of expression of the *HMA4* gene [25]. Therefore, HMA4-regulated Zn loading in roots is only one of the possible mechanisms determining intraspecific differences in the Zn accumulation capacity of *N. caerulescens*. Although the molecular mechanisms of Zn transport and detoxification in the Wilwerwiltz population are still largely unknown, it is clear that the increased transpiration rate at 500 µM Zn (Figure 2c) is only one of many possible factors that promote enhanced Zn accumulation in the shoots and particularly in the leaf epidermis.

Possible Zn detoxification mechanisms that might function more efficiently in Prayon compared to Wilwerwiltz include greater sequestration in the vacuoles due to higher density (as a result of higher gene copy number and/or expression) or activity of tonoplast transporters such as MTPs, HMA3, CAXs, MHX (a vacuolar Mg^2+^ and Zn^2+/^H^+^ exchanger), etc. [35,50,53] (see above), greater apoplastic compartmentation of Zn [95], and more effective Zn chelation by metal ligands, such as organic acids, amino acids, and metallothioneins [35,50,53]. For example, the highest expression levels of the *NcMT2a*, *NcMT2b*, and *NcMT3* genes encoding metallothioneins in the shoots of a superior metal-accumulating and hypertolerant calamine population of *N. caerulescens* from St-Laurent-le-Minier, compared to less tolerant and lower metal-accumulating populations La Calamine and Lellingen, suggest that metallothioneins contribute to the metal-adapted phenotype [118]. Treatment of *N. caerulescens* population from Plombières with Zn stimulated the accumulation of such organic acids as citrate and malate in leaves, whereas the accumulation of phytochelatins was not induced, suggesting that the accumulation of malate and citrate, but not the accumulation of phytochelatins, was responsible for Zn tolerance [119]. In general, in the shoots of the hyperaccumulators *N. caerulescens*, *A. halleri*, and *S. alfredii*, a low content of phytochelatins was observed, or they were completely absent [120]. Moreover, plant treatment with an inhibitor of the γ-glutamylcysteine synthetase—an enzyme in the biosynthetic pathway of glutathione, a precursor of phytochelatins—did not cause a reduction in the Zn tolerance of different populations of *N. caerulescens*, suggesting that phytochelatins and glutathione are not essential for Zn tolerance in this species [121]. Another mechanism that might underlie different Zn tolerance levels is the difference in the efficiency of the antioxidant defense system [36]. Though comparative studies of different populations of *N. caerulescens* in this regard are still lacking, Zn was shown to induce the activities of such antioxidant enzymes as superoxide dismutase, ascorbate peroxidase, guaiacol peroxidase and catalase in the leaves of *N. caerulescens*, though it highly and specifically depended on the Zn concentration in the medium [119].

### 4.5. Zinc-Induced Changes in the Contents of Mineral Elements

Mineral elements play a vital role in plant life [1]. Disruption of mineral nutrition is one of the most common manifestations of the toxic effects of Zn [2] and other potentially toxic elements [60,61]. Changes in the mineral composition of plants, also called the ionome [122], result from the interaction between the endogenous processes in plants and the effects of environmental factors [123,124]. Potentially toxic elements affect the absorption of other ions through multiple mechanisms, the relative role of which varies depending on the metal concentration, the physicochemical properties of the ions, as well as the plant species and their developmental stage. In most cases, potentially toxic elements inhibit the uptake and transport of both cations and anions, but for some elements, the interaction can be much more complex [2,60,61,124]. For example, Zn treatment led to an increase in the Fe content and a decrease in the Mn content in the roots of *T. aestivum* and *Beta vulgaris*, whereas the contents of both elements in the shoots decreased [13,15]. In *Saccharum* spp. plants, the Fe and Cu contents decreased, whereas the Mn content increased at high Zn concentrations in the growth medium [14]. In *N. caerulescens*, population from Røsos Copper Mine (Norway), exposed to 800 µM Zn for 10 days, the contents of Cu, Ca, Mg, Mn, and K in the roots as well as the contents of Mn and K in the shoots decreased, whereas the content of Cu in the shoots and the contents of Fe in the roots and shoots increased [125]. At the same time, the contents of Cu, B, and Mn in the shoots of the Bradford Dale population of *N. caerulescens* decreased, and the content of Fe therein slightly increased after 16 weeks of exposure to Zn (500 µM) [126]. Changes in the absorption of macro- and microelements under the influence of Zn can be determined by the similarity of the metal ionic radii, resulting in competition for common binding sites in transporters characterized by broad substrate specificity, such as, for example, IRT1 [53,99]. The existence of common transport pathways for Zn and a number of other elements was confirmed by significant correlations between the uptake, translocation, and accumulation of these elements (Table 2).

In the hydroponically grown Wilwerwiltz and Prayon plants, the differences in the contents of mineral elements were manifested in the roots and/or shoots, and, in the case of Fe, Mn, and Ca, these differences also depended on the Zn concentration in the nutrient solution (Figure 6, Figure 7, Figure 8, Figure 9, Figure 10 and Figure 11, Table 1). For example, the Mg content in the roots was similar in the Wilwerwiltz and Prayon plants, but the Mg content in the shoots was higher in the former, regardless of the Zn concentration in the medium (Figure 9a,b). In contrast, no differences in the Cu contents in the shoots were detected between the populations, whereas the Cu content in the roots was higher in the Wilwerwiltz plants regardless of the Zn concentration in the nutrient solution (Figure 8a,b). However, for the plants growing on soil, the patterns may differ for the reasons discussed above. Although not always significantly, non-metallicolous populations tend to have higher Mg, Ca, and Fe contents compared to metallicolous populations [64]. In the calamine edaphic group, the contents of K, phosphorus, and Cd in the shoots were higher, and the contents of Ca, Ni, and Zn were lower than those in the non-metalliferous and serpentine edaphic groups [41]. A possible explanation for the differences in mineral nutrient composition between the populations may be an increased expression or activity of relatively non-specific transporters involved in the uptake and/or root-to-shoot translocation of mineral nutrients [64].

The analysis of the obtained data revealed that the Zn-induced increase in the Fe content in the roots of both populations (Figure 6a) was due to an increase in its uptake (Figure 6c) and a decrease in its translocation (Figure 6d,e). These changes were more pronounced in Wilwerwiltz than in Prayon (Table 1), which caused a significant decrease in the Fe content in shoots only in the former (Figure 6b). The decrease in the chlorophyll content (Figure 3) and the signs of chlorosis that appeared in Wilwerwiltz at 500 µM Zn were associated mainly with the decrease in the Fe content, rather than the decrease in the Mg content (Figure 9), since the Mg content in Wilwerwiltz decreased only in the roots but increased in the shoots. The content of Ca in the roots increased in both populations (Figure 10a) due to a significant decrease in its translocation (Figure 10d,e), whereas Ca uptake decreased to a lesser extent (Table 1). As a result of decreased Ca uptake and translocation, its content in the shoots decreased in both populations (Figure 10b) with the increase in the Zn concentration in the medium. In contrast to Fe, the increase in the K content in the roots of Wilwerwiltz and Prayon (Figure 11a) resulted solely from the decrease in K translocation (Figure 11d,e), but not from the increase in its uptake (Figure 11c). For the other elements, clear intraspecific differences in Zn-induced changes were observed (Table 1). The Mn content in the roots of Wilwerwiltz did not change (Figure 7a) due to a decrease in both its uptake (Figure 7c) and its translocation (Figure 7d,e). In Prayon, an increase in the Mn content in the roots was observed (Figure 7a) due to a more intense decrease in its translocation (Figure 7d,e) than in its uptake (Figure 9b), with the latter still being less pronounced than that in Wilwerwiltz. The Cu content in the roots of Wilwerwiltz decreased (Figure 8a) due to a decrease in its uptake (Figure 8c), whereas no changes were observed in Prayon (Figure 8). Opposite changes in the Mg total uptake and content in the shoots were observed in the two populations (Table 1). In the Wilwerwiltz population, the Mg content in the roots decreased (Figure 9a), and its content in the shoots increased (Figure 9b), as a result of a greater increase in its translocation (Figure 9d,e) than in its uptake (Figure 9c). At the same time, the decrease in the Mg content in the roots (Figure 9a) and shoots of Prayon (Figure 9b) occurred due to a decrease in its uptake (Figure 9b), whereas its translocation did not change significantly (Figure 9d,e). In general, Zn-induced impairment of mineral nutrition was more pronounced in Wilwerwiltz than in Prayon (Figure 6, Figure 7, Figure 8, Figure 9, Figure 10 and Figure 11, Table 1), which resulted in the manifestation of Zn toxic effects in the former.

## 5. Conclusions

The Prayon population growing under natural conditions on Zn-enriched calamine soil is more tolerant to Zn than the Wilwerwiltz population naturally growing on non-metalliferous soil. The manifestation of the toxic effects of Zn, expressed as a decrease in the accumulation of root and shoot fresh and dry weights, a decrease in the water content in the roots and the contents of photosynthetic pigments in the shoots, as well as the appearance of signs of chlorosis, was observed at 500 µM Zn in the nutrient solution only in the Wilwerwiltz plants. The impairment of mineral nutrition under the action of Zn was more pronounced in Wilwerwiltz than in Prayon, which may be associated with the manifestation of Zn toxic effects in the former. The absence of the signs of Zn toxic effects in Prayon may be due to the lower Zn accumulation in Prayon than in Wilwerwiltz, as well as the more effective mechanisms of its detoxification. The higher Zn content in the shoots and, in particular, in the cells of the leaf epidermis in Wilwerwiltz than in Prayon may partly be determined by the higher transpiration rate in the former, at least at 500 µM Zn in the nutrient solution. These findings suggest that the metallicolous population maintains better control over Zn accumulation, which may be a part of the adaptive response to Zn-enriched medium. Further studies of the inter-population differences in hyperaccumulator species in terms of the efficiency of antioxidant defense system, metal effects on photosynthesis, respiration and other physiological processes, as well as the efficiency of metal transport across the plasma membrane and the tonoplast with the involvement of various transporters will contribute to our understanding of the mechanisms of metal tolerance and hyperaccumulation and will facilitate the use of these species in the technologies of phytoremediation and phytomining.

## Figures and Tables

**Table 1:** Zinc-induced changes in metal content, uptake, and translocation in the two populations of *Noccaea caerulescens* (Wilwerwiltz and Prayon).

Parameters	Fe		Mn		Cu		Mg		Ca		K	
	Wil	Pr	Wil	Pr	Wil	Pr	Wil	Pr	Wil	Pr	Wil	Pr
Metal content in roots	⇑ 9.1	⇑ 3.9	0	⇑ 1.8	⇓ 1.8	0	⇓ 1.2	⇓ 1.3	⇑ 1.9	⇑ 1.5	⇑ 1.2	⇑ 1.6
Metal content in shoots	⇓ 2.4	0	⇓ 3.5	⇓ 1.4	0	0	⇑ 1.8	⇓ 1.3	⇓ 1.2	⇓ 1.5	⇓ 1.1	⇓ 1.1
Total metal uptake	⇑ 1.4	⇑ 1.4	⇓ 3.3	⇓ 1.3	⇓ 1.4	0	⇑ 1.8	⇓ 1.3	⇓ 1.2	⇓ 1.5	0	0
Metal, % translocated	⇓ 3.3	⇓ 1.5	⇓ 1.1	⇓ 1.1	0	0	⇑ 1.1	0	⇓ 1.1	⇓ 1.1	⇓ 1.1	⇓ 1.1
Metal translocation factor	⇓ 23	⇓ 3.0	⇓ 2.6	⇓ 2.2	0	0	⇑ 2.2	0	⇓ 2.3	⇓ 2.2	⇓ 1.4	⇓ 1.8

An upward-facing arrow (⇑) and a number on a red background or a downward-facing arrow (⇓) and a number on a blue background indicate the fold increase or decrease, respectively, in the metal contents, uptake, or translocation in the plants from the Wilwerwiltz (Wil) and Prayon (Pr) populations along with the increase in the Zn concentration in the medium from 10 to 500 µM. Zero (“0”) indicates the absence of changes.

**Table 2:** Correlations between metal contents in roots, metal contents in shoots, total uptake of metals, and metal translocation factors, as well as between the percentage of translocated metals in the two populations of *Noccaea caerulescens* (Wilwerwiltz and Prayon). Given are the Pearson’s correlation coefficients (*r*). The significance of correlations is shown at the 0.05 (*), 0.01 (**), or 0.001 (***) levels. The intensity of pink color (for positive correlations) and blue color (for negative correlations) increases with the increase in the level of significance. The data for Wilwerwiltz (yellow heading) are above the grey diagonal and the data for Prayon (purple heading) are below the grey diagonal.

**Metal contents in the roots of the *Noccaea caerulescens* Wilwerwiltz population**								
	Zn	Fe	Mn	Cu	Mg	Ca	K	
Zn		0.946 ***	−0.409	−0.466	−0.745 **	0.814 ***	0.740 **	Zn
Fe	0.930 ***		−0.399	−0.472	−0.715 **	0.916 ***	0.595 *	Fe
Mn	0.860 ***	0.779 **		−0.209	0.277	−0.676 **	−0.454	Mn
Cu	−0.375	−0.402	−0.413		0.222	−0.413	−0.550 *	Cu
Mg	−0.768 **	−0.748 **	−0.470	0.546 *		−0.585 *	−0.302	Mg
Ca	0.846 ***	0.762 **	0.705 **	0.063	−0.513		0.596 *	Ca
K	0.901 ***	0.887 ***	0.740 **	−0.290	−0.698 **	0.824 ***		K
	Zn	Fe	Mn	Cu	Mg	Ca	K	
**Metal contents in the roots of the *Noccaea caerulescens* Prayon population**								
**Metal contents in the shoots of the *Noccaea caerulescens* Wilwerwiltz population**								
	Zn	Fe	Mn	Cu	Mg	Ca	K	
Zn		−0.776 **	−0.803 ***	0.035	0.923 ***	−0.510	−0.515	Zn
Fe	−0.091		0.722 **	0.365	−0.617 *	0.640 *	0.622 *	Fe
Mn	−0.776 **	0.244		−0.042	−0.769 **	0.401	0.423	Mn
Cu	−0.189	0.311	0.412		0.264	0.283	0.013	Cu
Mg	−0.721 **	0.006	0.782 ***	−0.001		−0.429	−0.490	Mg
Ca	−0.868 ***	0.261	0.626 *	0.430	0.469		0.053	Ca
K	−0.271	0.302	0.456	0.468	−0.035	0.385		K
	Zn	Fe	Mn	Cu	Mg	Ca	K	
**Metal contents in the shoots of the *Noccaea caerulescens* Prayon population**								
**Total metal uptake** **in the *Noccaea caerulescens* Wilwerwiltz population**								
	Zn	Fe	Mn	Cu	Mg	Ca	K	
Zn		0.707 **	−0.828 ***	−0.585 *	0.913 ***	−0.509	−0.049	Zn
Fe	0.681 **		−0.607 *	−0.669 **	0.634 *	−0.148	0.069	Fe
Mn	−0.764 **	−0.509		0.463	−0.795 ***	0.385	0.150	Mn
Cu	−0.095	0.203	0.217		−0.631 *	0.256	−0.218	Cu
Mg	−0.629 *	−0.554 *	0.611 *	−0.329		−0.425	−0.162	Mg
Ca	−0.899 ***	−0.522	0.606 *	0.258	0.503		−0.057	Ca
K	−0.062	0.084	0.150	−0.094	0.137	0.188		K
	Zn	Fe	Mn	Cu	Mg	Ca	K	
**Total metal uptake in the** ***Noccaea caerulescens* Prayon population**								
**Metal translocation factor** **in the *Noccaea caerulescens* Wilwerwiltz population**								
	Zn	Fe	Mn	Cu	Mg	Ca	K	
Zn		0.705 **	0.561 *	−0.133	−0.816 ***	0.683 **	0.772 **	Zn
Fe	0.899 ***		0.588 *	0.069	−0.711 **	0.899 ***	0.543 *	Fe
Mn	0.927 ***	0.876 ***		−0.478	−0.760 **	0.557 *	0.347	Mn
Cu	0.264	0.064	0.286		0.404	−0.201	−0.236	Cu
Mg	−0.512	−0.459	−0.541 *	0.083		−0.701 **	−0.665 **	Mg
Ca	0.717 **	0.664 **	0.563 *	0.406	−0.390		0.567 *	Ca
K	0.757 **	0.780 ***	0.852 ***	0.200	−0.532	0.856 ***		K
	Zn	Fe	Mn	Cu	Mg	Ca	K	
**Metal translocation factor in the** ***Noccaea caerulescens* Prayon population**								
**Percentage of metals translocated** **in the *Noccaea caerulescens* Wilwerwiltz population**								
	Zn	Fe	Mn	Cu	Mg	Ca	K	
Zn		0.872 ***	0.777 **	−0.199	−0.615 *	0.777 **	0.781 ***	Zn
Fe	0.926 ***		0.776 **	−0.356	−0.822 ***	0.859 ***	0.613 *	Fe
Mn	0.950 ***	0.926 ***		−0.389	−0.719 **	0.631 *	0.389	Mn
Cu	0.700 **	0.693 **	0.751 **		0.458	−0.361	−0.201	Cu
Mg	0.487	0.511	0.523	0.599 *		−0.666 **	−0.378	Mg
Ca	0.931 ***	0.913 ***	0.883 ***	0.818 ***	0.652 *		0.629 *	Ca
K	0.875 ***	0.862 ***	0.882 ***	0.853 ***	0.826 ***	0.959 ***		K
	Zn	Fe	Mn	Cu	Mg	Ca	K	
**Percentage of metals translocated in the** ***Noccaea caerulescens* Prayon population**								

## Data Availability

Data will be provided upon request.
